# The Effects of Green Tea Consumption on Cardiometabolic Alterations Induced by Experimental Diabetes

**DOI:** 10.1155/2012/309231

**Published:** 2012-02-29

**Authors:** Patricia Fiorino, Fabiana Sant'Anna Evangelista, Fernando Santos, Fátima Maria Motter Magri, Jan Carlo Morais O. B. Delorenzi, Milton Ginoza, Vera Farah

**Affiliations:** ^1^Renal, Cardiovascular and Metabolic Physiopharmacology Laboratory, Health and Biological Science Center, Mackenzie University, 01302-907 São Paulo, Brazil; ^2^School of Arts, Science and Humanities, University of Sao Paulo, Sao Paulo, Brazil

## Abstract

We evaluated cardiac autonomic modulation by heart rate (HRV), and arterial pressure variability (APV), and metabolic response in streptozotocin diabetic rats treated with green tea. Male Wistar rats were separated in groups: control, drinking tap water (C), green tea-treated (GT) group, diabetic, drinking tap water (D), and diabetic, treated with green tea (DGT). Kidney mass was greater in D and DGT than in C and GT, but reduced in DGT compared to D. Green tea prevented the increase in creatinine clearance and reduced hyperglycemia in DGT compared to D. Arterial pressure was increased in GT and decreased in D compared to C. HRV was reduced in D compared with all groups. APV was decreased in D compared to C and recovery in DGT. Sympathetic modulation of APV was decreased in D compared with all groups. Green tea reduced hyperglycemia, prevented renal injury and autonomic dysfunction, suggesting reduced cardiovascular risk and target organ damage in diabetes.

## 1. Introduction

 Diabetes mellitus (DM) is a metabolic disorder characterized by an impairment of carbohydrate, fat, and protein metabolism caused by either lack of insulin secretion or decreased sensitivity of the tissues to insulin [1]. This disease has reached epidemic levels in the United States and threatens a worldwide epidemic. The prevalence of diabetes is increasing rapidly, and the disease incidence in 2010 was about 285 million people worldwide, and is projected to increase to 438 million in 2030 [2].

 A number of studies have been published concerning the association between chronic hyperglycemia and cardiovascular complications in DM, including systemic hypertension, atherosclerosis, nephropathy, coronary ischemia, vascular disease, and stroke [3–7]. Dysfunction of autonomic neural control is a frequent complication of diabetes which is associated with high morbidity and mortality [8], and has been found in clinical and animal models of DM [9, 10]. Methods to detect early abnormalities in cardiac autonomic modulation in DM have been applied such as heart rate and arterial pressure variability analysis.

Heart rate and blood pressure variability (HRV and BPV), estimated in time or frequency (spectral analysis) domain, have been widely used to evaluate the cardiac autonomic modulation in both clinical and experimental studies [11–16]. Heart rate as well as blood pressure oscillations at low frequency (LF: 0.2 to 0.75 Hz) is an index of cardiac sympathetic modulation, while high-frequency (HF: 0.75 to 3 Hz) oscillations of HR reflect parasympathetic modulation of the heart [17]. The importance of HRV and BPV was established with reports that showed a reduction in HRV and/or increased in BPV is associated with a large number of pathologies like diabetes and cardiovascular diseases [12, 16, 18, 19]. Moreover, there is evidence showing that decrease in HRV is an independent predictor of sudden cardiac death as well as increased BPV was associated with end-organ damage [20–22].

Green tea (leaves of *Camellia sinensis*, Theaceae) is a popular beverage in east Asia, and in recent years green tea has been widely studied to assess its beneficial effects in the treatment and prevention of human disease [23, 24]. Several epidemiological studies and clinical trials suggest that green tea consumption reduces the risk of many chronic diseases, including cardiovascular diseases [23, 24]. Moreover, green tea consumption improved glucose metabolism implicated in DM [25, 26], revealing that green tea consumption could be beneficial in the management of DM. Additional published studies showed that the administration of green tea to STZ diabetic rats promoted blood glucose reduction, hypolipidemic response, antioxidant effect, improved kidney function, and cardiac protection [27–29]. However, only partial protection of green tea against STZ-induced hyperglycemia and oxidative stress has also been reported [30], suggesting that these contradictory results could be due to variations in severity, duration, and treatment of the disease.

Although the literature provides evidence that green tea is useful to DM treatment, no studies to date have tested the potential of green tea to prevent autonomic dysfunction associated with DM. Specifically, the present study evaluates the autonomic modulation of cardiovascular function as measured by heart rate, arterial pressure variability, and the metabolic response in STZ diabetic rats subject to green tea consumption since diabetes induction.

## 2. Materials and Methods

### 2.1. Animals

Experiments were performed on adult male (eight-week-old) Wistar rats weighing 260–300 g. The animals were randomly separated in four experimental groups: control, drinking tap water (C, *n* = 8), green tea-treated group (GT, *n* = 8), diabetic, drinking tap water (D, *n* = 9), and diabetic, treated with green tea (DGT, *n* = 10). The animals were housed in cages with free access to water and food, at a constant temperature of 23°C, on a 12-hour light/dark cycle. All experimental protocols were in accordance with the Guidelines for Ethical Care of Experimental Animals and were approved by the Institutional Animal Care and Use Committee (Protocol: 025/09/2008).

After the experimental period, rats were killed by over dose of sodium pentobarbital anesthesia (50 mg/Kg i.p.) and blood glucose, and creatinine concentrations were determined in nonfasting animals using colorimetric enzyme kits (LABTEST, Brazil). The kidneys were quickly removed and the ratios of kidney: body weight (kidney mass index) were determined.

### 2.2. Diabetes Induction

Diabetes was induced by a single injection of STZ (50 mg/kg, i.v., Sigma Chemical Co, St Louis, MO, USA) dissolved in 0.05 M citrate buffer, pH 4.5, administered 21 days before the treatment. Controls (C and GT) were injected with the vehicle (0.05 M citrate buffer, pH 4.5) alone. Animals were fasted for 4 h before STZ or vehicle injection. During the 24-hr after STZ induction (D and DGT) or vehicle injection (C and GT), the animals were fed with glucose solution (12.5 g/L) to avoid ketoacidosis.

Two days following STZ injection, diabetes induction was confirmed by blood glucose level (10 *μ*L of blood from tail vein) (Accu-Check Advantage Glucose Monitor, Roche Diagnostic Corporation, Indianapolis, IN, USA). Animals with blood glucose levels less than 250 mg/dL or greater than 400 mg/dL were excluded from the study to avoid great discrepancies in our data due to changes in glucose levels since it is well known the influence of glucose levels on metabolic and cardiovascular functions.

### 2.3. Green Tea Preparation, Analysis, and Treatment

Green tea was prepared daily by adding 3.0 g of dry green tea (Farmanatural, Natural Pharma, São Paulo, Brazil) to 1000 mL of boiled water cooled to 90°C. The solution was filtered after 15 min, cooled to room temperature, and dispensed into clean drinking bottles. The total content of caffeine and polyphenols in the green tea solution was 0.07% and 422.80 *μ*g/mL, respectively. Green tea solution was administered for 21 days to the GT and DGT groups.

### 2.4. Metabolic Cages

In a subgroup of rats randomly selected (C, *n* = 7; GT, *n* = 6; D, *n* = 6; DGT, *n* = 8), at the end of the second week (day 14), animals were housed separately for 48 h in metabolic cages (Andrade's, São Paulo, Brazil) with free access to food and tap water or green tea solution. Hydric consumption, urinary excretion, and food intake during the last 24 h between 9:00 am and 10:00 am. Urine glucose and creatinine were measured using a colorimetric enzyme assay (LABTEST, Brazil).

### 2.5. Glucose Tolerance Test (GTT)

At the end of the study, in a subgroup of randomly selected rats (C, *n* = 7; GT, *n* = 5; D, *n* = 7; DGT, *n* = 5), glucose was measured using an Accu-Check Advantage Blood Glucose Monitor (Roche Diagnostic Corporation, Indianapolis, IN, USA). Animals were fasted for 4 h, given an intraperitoneal glucose load (1.5 g/kg, i.p.), and blood samples were taken at baseline and 15, 30, 60, and 90 min from a cut made on the tip of the tail.

### 2.6. Cardiovascular Measurements

One day prior to arterial pressure (AP) recordings, an arterial catheter was placed in the right femoral artery of study animals under ketamine-xylazine anesthesia (50 : 10 mg/kg i.p.), for the direct measurement of AP in all groups (C, *n* = 8; GT, *n* = 8; D, *n* = 9; DGT, *n* = 10). The catheter was exteriorized through the back of the neck.

During the study, AP was recorded during 30 minutes via a transducer (Hewlett-Packard 1280, USA) connected to the arterial catheter. Animals were conscious and moved freely during recording. The AP signal was fed into an amplifier (GPA-4 model 2, Stemtech Inc.) connected to a 16-channel analog-to-digital interface, and continuously sampled (2 kHz) on an IBM/PC (T23, IBM Thinkpad, Inc). Beat-to-beat values of systolic, diastolic, and mean AP (SAP, DAP and MAP, resp.) were determined, and heart rate (HR) or pulse interval (PI) were calculated as the interval between successive systolic pressure values using the software application WinDaq (DataQ Instruments, Inc., USA).

PI and SAP fluctuations were assessed in the time and frequency domains using autoregressive spectral analysis, as described elsewhere [12, 15, 31, 32]. The theoretical and analytical procedures for autoregressive modeling of oscillatory components have been fully described previously [12, 13, 17]. Briefly, the PI and SAP series derived from each recording were divided into 300-beat segments with a 50% overlap. The spectra of each segment were calculated via the Levinson-Durbin recursion, and the order of the model chosen according to Akaike's criterion, with the oscillatory components quantified in low-(LF: 0.2 to 0.6 Hz) and high-frequency (HF: 0.6 to 3.0 Hz) ranges.

### 2.7. Statistical Analyses

Data are reported as mean ± SEM. Statistical analyses were performed using two-way analysis of variance (ANOVA) followed by the Bonferroni test. Differences were considered to be significant at *P* < 0.05.

## 3. Results

### 3.1. The Effect of Green Tea on Metabolic Measurements

Table 1 shows the effect of diabetes and green tea consumption on body weight and kidney mass index. No differences in body weight were observed among groups in the beginning of the experiment. However, both diabetic groups (D and DGT animals) showed lower body weight compared to nondiabetic control groups (C and GT animals) after the period of 21 days (*P* < 0.05). There was no significant difference between the mean body weights of the D group compared with DGT showing that green tea consumption did not prevent weight loss in diabetic animals. In fact, while the groups C and GT showed a significant increase in body weight (20% and 19%, resp., *P* < 0.05), a significant reduction in body weight was observed in D (7.5%) and DGT (9.6%) groups at the end of the study. The kidney mass index was greater in D and DGT than in C and GT animals (*P* < 0.05). However, green tea intake reduced the kidney mass index in DGT animals compared with D (5.0 ± 0.2 versus 6.3 ± 0.2 mg/g, resp., *P* < 0.05).

**Figure 1:**
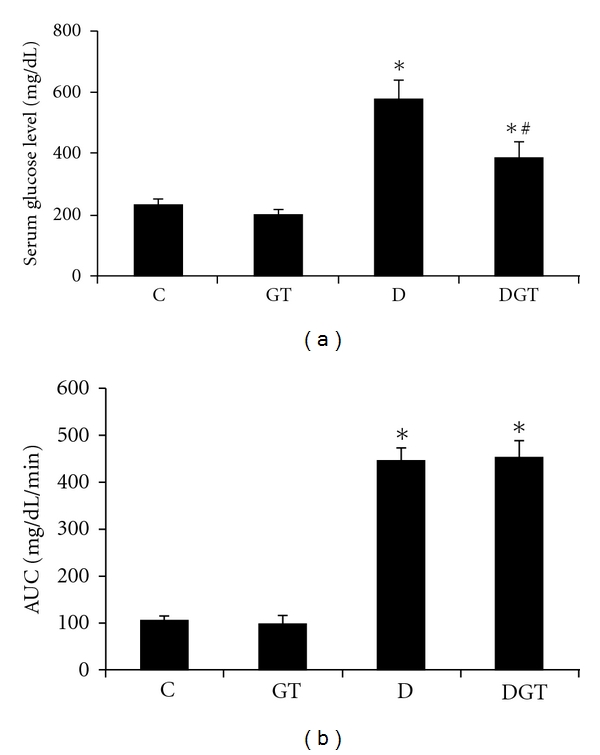
Serum glucose concentration (a) and area under curve (AUC) of blood glucose (b) during glucose tolerance test. C: control, drinking tap water group; GT: green tea-treated group; D: diabetic, drinking tap water; DGT: diabetic, treated with green tea group. Serum glucose: C (*n* = 8), GT (*n* = 8), D (*n* = 9), and DGT (*n* = 10). Glucose tolerance test: C (*n* = 7), GT (*n* = 5), D (*n* = 7), and DGT (*n* = 5). GTT: C (*n* = 7), GT (*n* = 5), D (*n* = 7) and DGT (*n* = 5). * *P* < 0.05 versus C and GT; ^**#**^ *P* < 0.05 versus D.

The induction of diabetes mellitus in the experimental animals was confirmed by blood glucose value above 250 mg/dL 48 h after STZ induction. D and DGT animals developed hyperglycemia (577 ± 62 mg/dL and 384 ± 52 mg/dL, resp.), their serum glucose concentrations being significantly higher than nondiabetic animals (C, 232 ± 16 mg/dL and GT, 202 ± 16 mg/dL). Diabetic rats treated with green tea (DGT) showed lower hyperglycemia than diabetic rats not treated with green tea (D), suggesting that green tea consumption protects from severe hyperglycemia in diabetic rats. It is important to note that green tea did not change the serum glucose level in GT animals (Figure 1(a)). Despite hyperglycemia suppression by green tea, we observed no differences in the area under curve (AUC) of the glucose tolerance tests between D (446 ± 29 mg/dL/min) and DGT (454 ± 39 mg/dL/min), nor between C (109 ± 9 mg/dL/min) and GT (101 ± 18 mg/dL/min) groups, confirming that green tea did not change glucose tolerance in STZ-induced diabetic rats (Figure 1(b)). 

 As shown in Table 1, diabetic animals (D and DGT) showed signs of polydipsia, polyphagia, and polyuria. No significant differences between diabetic groups (D versus DGT) nor between control animals (C versus GT) were observed. The administration of green tea did not exert a significant effect on water or food intake as well as in urine volume collected in metabolic cages over 24 hours. However, it is important to note that DGT animals ingested higher amount of green tea (0.3  ± 0.05 g/day) than GT animals (0.06  ± 0.01 g/day). Glomerular filtration rate assessed by creatinine clearance (Table 2) was increased in diabetic (D and DGT) compared with nondiabetic rats C and GT (*P* < 0.0001). Green tea consumption prevented the strong increase in creatinine clearance in the DGT group (0.75 ± 0.1 mL/min/Kg) compared with D group (1.1 ± 0.3 mL/min/Kg). However, in nondiabetic rats there was no statistically significant effect of green tea on creatinine clearance (0.21 ± 0.03 versus 0.43 ± 0.09 mL/min/Kg, C and GT resp.). Finally, diabetic rats treated with green tea showed a significant reduction in urine glucose compared with D. Although, there was a difference between C and GT in urine glucose, it was not statistically significant (Table 2).

### 3.2. The Effect of Green Tea on Cardiovascular Measurements

The values obtained for MAP and HR are presented in Table 3. Diabetes induced by STZ promoted attenuation in MAP (~10% of reduction) as well as in HR (~20% of reduction) in D group. Green tea intake prevented the reduction of both parameters, MAP and HR in diabetic animals. However, in nondiabetic animals green tea increased MAP (~20% of increase) with no alterations in HR. The total power of the HRV was reduced in D in comparison with all groups. The APV decrease in D compared to C and recovery in DTG. Sympathetic modulation of the arterial pressure variability (LF) decreased in D compared with groups C, GT, and DGT.

## 4. Discussion

The present study was conducted to evaluate the role of green tea consumption in metabolic response and cardiovascular autonomic modulation in STZ-induced diabetic rats. Experimental diabetes induced by STZ is a well-established method used to evaluate the mechanisms involved in the alterations of physiopathology observed in diabetic patients. STZ destroys pancreatic b cells, resulting in a diabetic syndrome in animals, similar to that seen in human type 1 diabetes and characterized by hyperglycemia, hypoinsulinemia, glucosuria, and loss in body weight [33–35]. 

Although the nondiabetic rats registered approximately 20% growth in body weight, diabetic rats showed reduced body weight and green tea extract treatments did not improve weight gain in STZ-treated animals. These responses were also observed by Renno et al. [29] and Juśkiewicz et al. [30]. In other studies, the consumption of green tea prevented the loss in body weight in STZ-treated rats [27, 28]. However, the contradictory results could be due to the differences in dosage and methods used in dietary treatment. Despite green tea plays an important role in the regulation of body weight [36], our results showed that the body weight of nondiabetic rats, C and GT, was similar. This response may be associated with the lower content of caffeine in the green tea extract (0.07%) used in the present study, if we considered that the caffeine is the main factor involved in thermogenesis, fat oxidation, and sparing fat-free mass [37].

In the present study, diabetic-treated (DGT) and untreated (D) animals showed serum, and urine glucose levels elevated compared with C group. However, we observed a significant reduction in both serum and urine glucose levels in the DGT group as compared to the D group. Considering that hyperglycemia is the principal factor responsible for kidney and cardiovascular damage [7], the antihyperglycemic effect of green tea observed in the DGT group suggests an important clinical relevance to diabetes treatment. In support of the present results, recent reports have shown that green tea administration caused similar antihyperglycemic effect in STZ induced rats [28, 29], in diabetic db/db mice [25] and in diabetes type 2 patients [38]. On the other hand, the effect of green tea on glucose tolerance was not observed in both nondiabetic (GT) and diabetic (DGT) groups. Similar results with glucose tolerance were shown by Wu et al. [39].

With regard to metabolic parameters, STZ rats showed polydipsia, polyphagia, and polyuria [27]. However, the administration of green tea in this study did not exert a significant effect on liquid intake or food intake as well as in urine volume since no significant differences among groups were observed. Despite our results were similar to that showed by Tomlinson et al. [34], Babu et al. [27] observed decreased food and water intake in diabetic rats treated with green tea. The differences may be associated with the period and moment that green tea treatment was introduced in STZ rats. In the present study, green tea was administrated for 21 days following the first day of diabetes identification while in the other study green tea was administrated for 4 weeks and the treatment was begun 6 weeks after the onset of diabetes [27].

Glomerular filtration rate assessed by creatinine clearance was measured in our study to provide information about renal function. Diabetic rats (D and DGT) increased creatinine clearance compared with nondiabetic rats C and GT. It is interesting that green tea consumption resulted in lower creatinine clearance in DGT compared with D, but did not affect nondiabetic rats (C versus GT), thus, our data suggests that green tea prevents glomerular hyperfiltration in diabetes. In support with our data, it has been reported that in rats with streptozotocin-induced diabetes, green tea consumption showed a significant reduction in renal injury associated with hyperglycemia [29].

Diabetes caused an abnormal increase in kidney mass index in both diabetic groups D and DGT. Interestingly, green tea consumption by DGT group significantly reduced diabetes-induced hypertrophy of kidney by 20% as compared to the D group. These results suggest the importance of green tea in reducing kidney hypertrophy and corroborate another study with STZ rats [30]. Creatinine clearance as well as kidney mass are important markers of nephropathy and our data are in agreement with Ribaldo et al. [40] that showed a decrease in the markers of nephropathy in diabetic hypertensive rats following the consumption of green tea.

In the present study, we observed in rats with STZ-induced diabetes a reduction in AP and HR associated with a decrease in HRV and APV as previously described [10, 41, 42]. On the other hand, there are reports of increased blood pressure [43–45] or no change in STZ-induced diabetic rats [46]. Some of discrepancies in the literature regarding BP changes in STZ rats may be due to differences in age and time of experimentation as well as the methodology for BP measurement. However, the new data is that green tea consumption was able to prevent all of these cardiovascular alterations. Since the HRV and APV are useful tools to evaluate autonomic modulation of the cardiovascular system in humans and experimental models, our data suggests that green tea consumption was able to reduce the autonomic neuropathy observed in diabetes. It is well accepted that diabetes is associated with elevated oxidative stress [47, 48], which is correlated with alterations in the autonomic control of the circulation [14, 49].

The favorable effects attributed to the green tea extract in the prevention of cardiovascular diseases are correlated with the antioxidant properties of the catequins, which are the major components of green tea [50]. Experimental studies have been demonstrated the increase in total plasma antioxidant activity determined by green tea catequins [51, 52]. Moreover, *in vitro* studies have demonstrated that green tea extracts and tannin mixtures have a direct scavenging activity against nitric oxide and superoxides [53]. Thus, we can hypothesize that antioxidants, such as green tea may have beneficial effects on the cardiovascular autonomic nervous system. In fact, previous studies showed that the administration of antioxidants, like vitamin E, exerts beneficial effects on the cardiac control by rebalancing autonomic nervous system in diabetes [54]. Therefore, when taken together, these data support the conclusion that other studies need to be carried out to better understand the mechanisms involved in green tea pharmacological action and also reveal that indiscriminate chronic consumption of green tea might be a risk for health individuals, since in our study we have observed that the green tea ingestion determined an increase in blood pressure in GT.

In summary, the major findings of this study are that green tea decreased hyperglycemia and prevented renal injury by improvement of glomerular filtration and kidney hypertrophy observed in diabetic rats. Moreover, green tea was able to prevent the autonomic dysfunction in diabetic rats by blocking the alterations in arterial pressure variability. In conclusion, our data suggest that green tea consumption has important effects in reducing the cardiovascular risk and in targeting organ damage observed in diabetes.

## Figures and Tables

**Table 1:** Body weight, kidney mass index, and parameters obtained of metabolic cage.

	C	GT	D	DGT
Initial body weight (g)	280 ± 6	270 ± 9	265 ± 4	260 ± 8
Final body weight (g)	335 ± 14.0	320 ± 11	245 ± 14*	235 ± 18*
Left kidney mass index (mg/g)	3.7 ± 0.2	4 ± 0.2	6.3 ± 0.2*	5.0 ± 0.2^∗#^
Right kidney mass index (mg/g)	3.6 ± 0.1	4.3 ± 0.2	6.5 ± 0.2*	5.2 ± 0.2^∗#^
Food intake (g)	15 ± 1.6	11 ± 2.5	23 ± 6.6*	31 ± 4*
Hydric consumption (mL/24 h)	19 ± 1.7	21 ± 2.6	73 ± 4.6*	90 ± 15*
Urinary excretion (mL/24 h)	6 ± 0.6	8 ± 0.7	61 ± 12*	77 ± 12*

C: control, drinking tap water group; GT: green tea-treated group; D: diabetic, drinking tap water; DGT: diabetic, treated with green tea group. Initial and final body weight (g) and kidney mass index (mg/g): C (*n* = 8), GT (*n* = 8), D (*n* = 9), and DGT (*n* = 10). Metabolic cage: C (*n* = 7), GT (*n* = 6), D (*n* = 6) and DGT (*n* = 8). *P < 0.05 versus C and GT; ^#^ *P* < 0.05 versus D.

**Table 2:** Changes in urine, serum glucose, and creatinine clearance after 21 days of treatment with green tea in diabetic rats induced by streptozotocin.

Groups	C	GT	D	DGT
Urine glicose (mg/dL)	3 ± 1.7	14 ± 3.2	845 ± 94*	637 ± 58^∗#^
Serum glicose (mg/dL)	232 ± 16	202 ± 16	577 ± 62*	384 ± 52^∗#^
Creatinine clearance (mL/min/Kg)	0.21 ± 0.03	0.43 ± 0.09	1.1 ± 0.3*	0.75 ± 0.1^∗#^

C: control, drinking tap water group; GT: green tea-treated group; D: diabetic, drinking tap water; DGT: diabetic, treated with green tea group. Serum glucose: C (n=8), GT (n=8), D (*n* = 9), and DGT (*n* = 10). Urine glucose: C (*n* = 7), GT (*n* = 6), D (*n* = 6), and DGT (*n* = 8). *P < 0.05 versus C and GT; ^#^ *P* < 0.05 versus D.

**Table 3:** Mean arterial pressure, heart rate, and respective variabilities in time and frequency domains in diabetic rats subject to green tea treatment.

	C (*n* = 8)	GT (*n* = 8)	D (*n* = 9)	DGT (*n* = 10)
MAP(mmHg)	106 ± 2	128 ± 3^#^	99 ± 1.5*	111 ± 4
HR (bpm)	360 ± 7	341 ± 8	295 ± 11*	328 ± 14
HRV (ms^2^)	18 ± 1.3	16 ± 1.1	8.8 ± 0.7*	15 ± 1.6
APV (mmHg^2^)	23 ± 3.2	31 ± 7.5	7.5 ± 0.2*	15 ± 4.4
LF (mmHg^2^)	2.5 ± 0.36	3.8 ± 0.6	0.9 ± 0.4*	3.1 ± 0.8

C: control, drinking tap water group; GT: green tea-treated group; D: diabetic, drinking tap water; DGT: diabetic, treated with green tea group. MAP: mean arterial pressure; HR: heart rate; HRV: heart rate variability; APV: arterial pressure variability; LF: low-frequency domain of APV. *P < 0.05 versus all groups; ^#^ *P* < 0.05 versus C.
